# Assessment of the level III of Inoue by preoperative endoscopic ultrasound and elastography: a novel approach to predict a periarterial divestment technique in borderline resectable (BR) or locally advanced (LA) pancreatic adenocarcinoma—How I do it

**DOI:** 10.1007/s00423-023-03105-x

**Published:** 2023-09-21

**Authors:** Piero Alberti, Elizabeth Pando, Monder Abu-Suboh, Miquel Masachs, Xavier Merino, Maite Salcedo, Teresa Macarulla, Joaquin Balsells, Ramon Charco

**Affiliations:** 1 Department of Surgery, Universitat Autonoma de Barcelona, Barcelona, Spain; 2 Department of Hepatopancreatobiliary and Trasplant Surgery, Hospital Vall d’Hebron, Barcelona, Spain; 3 Digestive Endoscopy Unit, Hospital Vall d’Hebron, Barcelona, Spain; 4 Radiology Department, Hospital Vall d’Hebron, Barcelona, Spain; 5 Anatomopathology Department, Hospital Vall d’Hebron, Barcelona, Spain; 6 Oncology Department, Hospital Vall d’Hebron, Vall d´Hebrón Institute of Oncology, Barcelona, Spain

**Keywords:** Periarterial divestment, Pancreatic cancer, Locally advanced pancreatic cancer, Triangle operation, Borderline pancreatic cancer

## Abstract

**Background:**

Periarterial divestment is a surgical technique to approach borderline resectable (BR) or locally advanced (LA) pancreatic ductal adenocarcinoma (PDAC) with arterial involvement. There are no reports in the literature regarding the role of endoscopic ultrasound and elastography (EUS-EG) in exploring the integrity of Inoue’s level III and its correlation with the periarterial divestment technique feasibility. Our research is aimed at exploring the role of EUS-EG in this scenario.

**Methods:**

We describe our approach to Inoue’s level II by EUS-EG in patients with BR and LA pancreatic cancer patients after neoadjuvant chemotherapy.

**Results:**

Between June 2019 and December 2020, four patients out of 25 were eligible to perform a preoperative EUS-EG. In all cases, Inoue’s level III integrity was corroborated by EUS-EG and confirmed posteriorly in the surgical scenario where a periarterial divestment technique was feasible. Vein resections were necessary in all cases, with no need for arterial resection. An R0 (> 1 mm) margin was achieved in all patients, and the histopathological assessment showed the presence of neurovascular tissue at the peripheral arterial margin.

**Conclusion:**

Preoperatively, EUS-EG is a novel approach to explore the integrity of Inoue’s level III and could be helpful to preclude a periarterial divestment technique in borderline resectable or locally advanced pancreatic adenocarcinoma with arterial involvement.

## Introduction

Borderline resectable (BR) or locally advanced (LA) pancreatic ductal adenocarcinoma (PDAC) with arterial involvement remains a challenge for surgical resectability [1]. In the era of neoadjuvant therapies (NT), the proportion of patients who can undergo resection leading to better prognosis and long-term survival has increased substantially [2]. A systematic review reported a resectability rate of 28% after NT in patients with LAPC [3].

Periarterial divestment is a surgical technique to approach BR or LA with arterial encasement after NT by dissecting the periadventitial space between the tumour and arterial vessel, following level III of the mesopancreas, described by Inoue [4]. Additionally, dissection through the neurovascular level III facilitates an ‘*en-bloc*’ resection of the right semicircle of the superior mesenteric artery (SMA)-nervus plexus, obtaining cancer-free margins, avoiding arterial resections, which has been associated with high perioperative morbidity and mortality [1]. Periarterial divestment is possible when the tumour is not infiltrating across the Inoue’s level III to the adventitia; consequently, an arterial infiltration is absent.

Endoscopic ultrasonography (EUS) is used to assess vascular infiltration in pancreatic cancer, and its association with elastography (EUS-EG) has been found to increase the diagnostic power by evaluating hardness, softness, and the relative movement of two tissues at the same compression level [5]. Nevertheless, there are no reports in the literature regarding the role of EUS-EG in exploring the integrity of Inoue’s level III and its correlation with the surgical findings and periarterial divestment technique feasibility.

Thus, we described our experience using EUS-EG as an approach towards arterial involvement in the settings of BR and LA pancreatic cancer after NT in order to assess the integrity of the level III of Inoue, corroborated in the surgical scenario with the hypothetical benefit to be a preoperative diagnostic technique.

This study is aimed at showing our first case series following this novel approach.

## Methods

We describe our technique to approach the integrity of Inoue’s level III preoperatively using EUS-EG by performing a case series report of patients with BR or LA pancreatic cancer after completing neoadjuvant chemotherapy. All patients are discussed in the pancreatic cancer multidisciplinary board from our hospital, a third-level reference hospital in pancreatic cancer. Our centre performed around 60 pancreatic resections/year and at least 720 EUS-EG/year.

### Inclusion criteria

Inclusion criteria are patients with a RECIST 1.1 (Response Evaluation Criteria in Solid Tumours) [6] regression or stable disease criteria who presents a critical arterial encasement of the common hepatic artery (CHA), superior mesenteric artery (SMA), or celiac trunk (CT), with suspicion of infiltration in the computed tomography scan (more than 90° around arterial vessels) who are candidates to an exploratory laparotomy.

### Exclusion criteria

Exclusion criteria are patients with isolated venous involvement; arterial contact of less than 90°; proper hepatic artery involvement, patients with infiltration of CT in whom a resection (Modified Apple by) is proposed; patients not candidates for an exploratory laparotomy: (1) patients with LAPC with extended arterial involvement, considered LAPC unresectable (LAPC type III) [7] with clear signs of arterial infiltration on concomitant CT, CHA, and SMA, and patients with a non-reconstructible portal or mesenteric vein.

### BR and LA pancreatic cancer definitions

Borderline resectable or locally advanced pancreatic adenocarcinoma is defined according to the NCCN radiological definition at the time of diagnosis based on the computed tomography multiphase scan [8].

### Neoadjuvant chemotherapy

Neoadjuvant chemotherapy consists of FOLFIRINOX in < 75 years old and fit patients, or gemcitabine alone, or gemcitabine plus Nab-paclitaxel when the patient is not fit enough to be treated with FOLFIRINOX. For BR and LA, the treatment duration of neoadjuvant chemotherapy is at least 8 cycles for FOLFIRINOX and 4 cycles for gemcitabine ± Nab-paclitaxel.

### Computed tomography scan and CA 19–9 evaluation

CT scan and CA 19–9 levels are done at diagnosis, 2 months after neoadjuvant treatment and at the end of treatment. The CT scan protocol consists of a triphasic protocol with a late arterial phase (35–40 s after intravenous contrast injection) and a late portal phase (60–70 s after intravenous contrast injection). A complete laboratory test work-up includes CA 19–9 and CEA levels. Patients are discussed with a multidisciplinary board of pancreatic cancer at diagnosis every 2 months and when NT is finished. CT scans are reviewed by two experts radiologist from our multidisciplinary board.

### *Endoscopic ultrasound* + *elastography evaluation*

i) Patients who meet inclusion and exclusion criteria are eligible to perform an EUS-EG.
ii) EUS-EG is performed the day before surgery by the same experienced endoscopist to evaluate the integrity of Inoue’s level III. Technical considerations include a radial EUS scope, GF-UE 160-AL5 Olympus, an Olympus EUS processor, and EU-ME2. The procedure was carried out at the clinic under monitored sedation.
iii) EUS-EG protocol includes an assessment of pancreatic tumour and their relationship with vessels:
  1. For vessels: (a) grade of vessel contact: < 90/90–180, 180–270, > 270; (b) deformity (irregularity): yes/no; (c) lumen reduction: no, < 50%, > 50%, occlusion; (d) thrombus in the vessel: yes/no; (e) length of stenosis in mmm.
  2. For Inoue’s level III evaluation, a hyperechogenic line between the vessel wall and tumour suggests the integrity of Inoue’s level. Additionally, the presence of green lines on elastography around vessels indicated the integrity of the vascular wall.

### Surgical technique

In our centre, we perform an SMA artery first approach [9] to determine resectability first.

Independently of the EUS-EG evaluation results, we follow an intending to treat principle. When arterial involvement is suspected, we reproduce the periarterial divestment technique previously described by experienced centres [10] to release the tumour from the arterial vessel, avoiding arterial resection. When a periadventitial dissection is not feasible, we abort the procedure and perform a palliative by-pass (biliary or gastric) if necessary. In fit patients under 65 years old with no significant comorbidities, an arterial resection with anastomosis is offered contrary to a palliative procedure.

A partial (180°) or total circumferential excision of the SMA-mesopancreas, according to the degree of arterial involvement, is performed. Regional lymphadenectomy is performed as routine. Venous reconstruction is performed according to the type of defect (tangential, terminal-terminal, peritoneum patch, cadaveric graft, or PTFE graft if necessary).

After the surgical specimen extraction, the arterial and venous bed is marked to facilitate the margin evaluation for the pathologist. The pathological evaluation is performed by an experienced pathologist in pancreatic tumours using the axial slicing technique [10]. We consider an R0 resection when a free margin ≥ 1 mm was achieved.

### Statistics and ethics

Descriptive statistics were performed. Our local ethical committee approved our prospective data register following the Helsinki Declaration principles for human investigations. All patients signed informed consent for both EUS-EG and surgical intervention.

## Results

Between June 2019 and December 2020, four patients of 25 were eligible to perform a preoperative EUS-EG according to the inclusion and exclusion criteria. Excluded patients were as follows: thirteen patients presented with venous contact only, three patients were excluded due to less than a 90° SMA, one patient had 180° CT contact and was proposed for a modified Apple By procedure (arterial divestment technique was not the chosen technique), one patient had less than a 90° CT contact, and four patients had proper hepatic artery involvement.

The mean age was 52 years, and three were female. Due to a delay in the surgical schedule because of the COVID-19 pandemic, patients A and B received additional cycles of neoadjuvant chemotherapy than initially planned, and patient A underwent radiotherapy. Patient C was included in a clinical trial, limited to receiving no more than five cycles of FOLFIRINOX. Patient D received eight cycles of FOLFIRINOX according to our routine clinical practice. Tumour marker Ca 19–9 decreased > 50% of its initial value in three patients, except in one case that remained stable. Patient’s and tumour characteristics are listed in Table 1.

**Table 1:** Patients and tumour characteristics

Case	Age	Sex	ASA/ECOG	BMI (kg/m2)	Pathological history	Neoadjuvant therapy (#cycles) + RT (grays)	NCCN criteria at diagnostic		RECIST criteria	NCCN criteria post neoadjuvant therapy		EUS + EG	Ca 19–9 (U/ml) pre-neoadjuvant therapy	Ca 19–9 (U/ml) post-neoadjuvant therapy
							Artery	Vein		Artery	Vein			
A	53	Male	2/0	26.9	Type 2 diabetes. Exploratory laparotomy in other centre (no resection possible)	FOLFIRINOX (11) + RT(54 Gy)	180° SMA	SMV encasement	Regression	< 180° SMA	SMV encasement	Integrity of Inoue’s plane 3	90	30
B	54	Female	2/0	21.9	None	NALIRINOX (12)	< 180° HA + < 180° SA	Porto-splenic confluence encasement	Stable	< 180° HA + < 180° SA	Porto-splenic confluence encasement	Integrity of Inoue’s plane 3	206	21
C	53	Female	2/0	30.3	None	FOLFIRINOX (5)	SMA > 270°, CT 180°–270°, CHA 90°–180°	SMV > 270°, PV 90°–180°	Stable	SMA > 270°, CT 180°–270°, CHA 90°–180°	SMV > 270°, PV 90°–180°	Integrity of Inoue’s plane 3	370	370
D	47	Female	2/0	27.4	None	FOLFIRINOX (8)	> 180° SMA	Porto-mesenteric confluence encasement	Regression	< 180° SMA	< 270° SMV	Integrity of Inoue’s plane 3	8744	140

*ASA*, American Society of Anaesthesiologists; *ECOG*, Eastern Cooperative Oncology Group; *BMI*, body mass index; *FOLFIRINOX*, fluorouracil, irinotecan, and oxaliplatin; *NALIRINOX*, nanoliposomal irinotecan plus fluorouracil/leucovorin; *RT*, radiotherapy; *SMA*, superior mesenteric artery; *HA*, hepatic artery; *SA*, splenic artery; *CT*, celiac trunk; *CHA*, common hepatic artery; *SMV*, superior mesenteric vein; *PV*, portal vein; *RECIST*, Response Evaluation Criteria in Solid Tumours; *NCCN*, National Comprehensive Cancer Network; *EUS*, endoscopic ultrasound

### CT scan findings

**Fig. 1:**
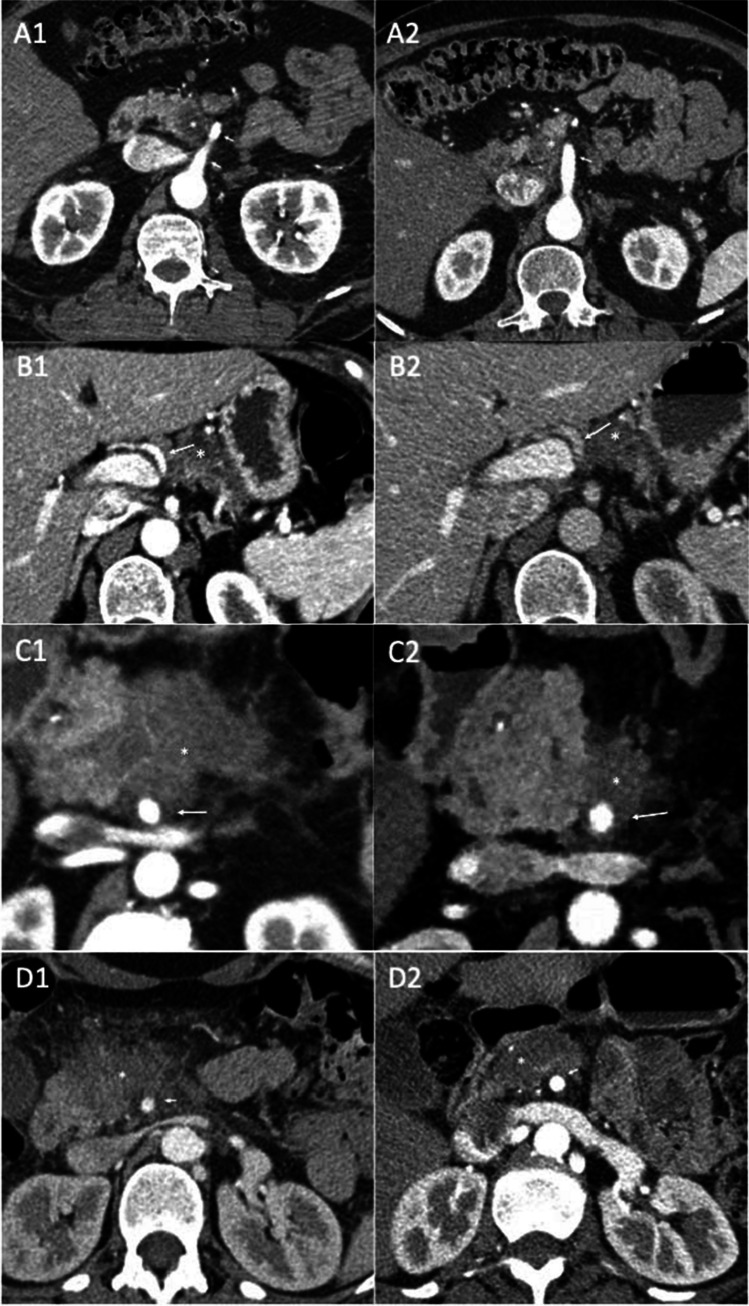
Cases A, C, and D show tumour contact with SMA before (**A1**, **C1**, and **D1**) and after NT (**A2**, **C2**, and **D2**). Case B shows tumour contact with HA before (**A1**) and after NT (**A2**). No separation plane between the tumour and vessel wall is seen. NT, neoadjuvant treatment; SMA, superior mesenteric artery; HA, hepatic artery

Patients showed a partial response after NT in 2 cases, and 2 showed stable disease in CT scans (Fig. 1). Regarding the relation with arterial margins, the CT scan showed the persistence of tumour contact around vessels, with no signs of deformity or irregularity. Based only on the CT scan findings, it was not possible to determine if a potential divestment technique between the tumour and the adventitial plane could be performed.

### EUS-EG results

**Fig. 2:**
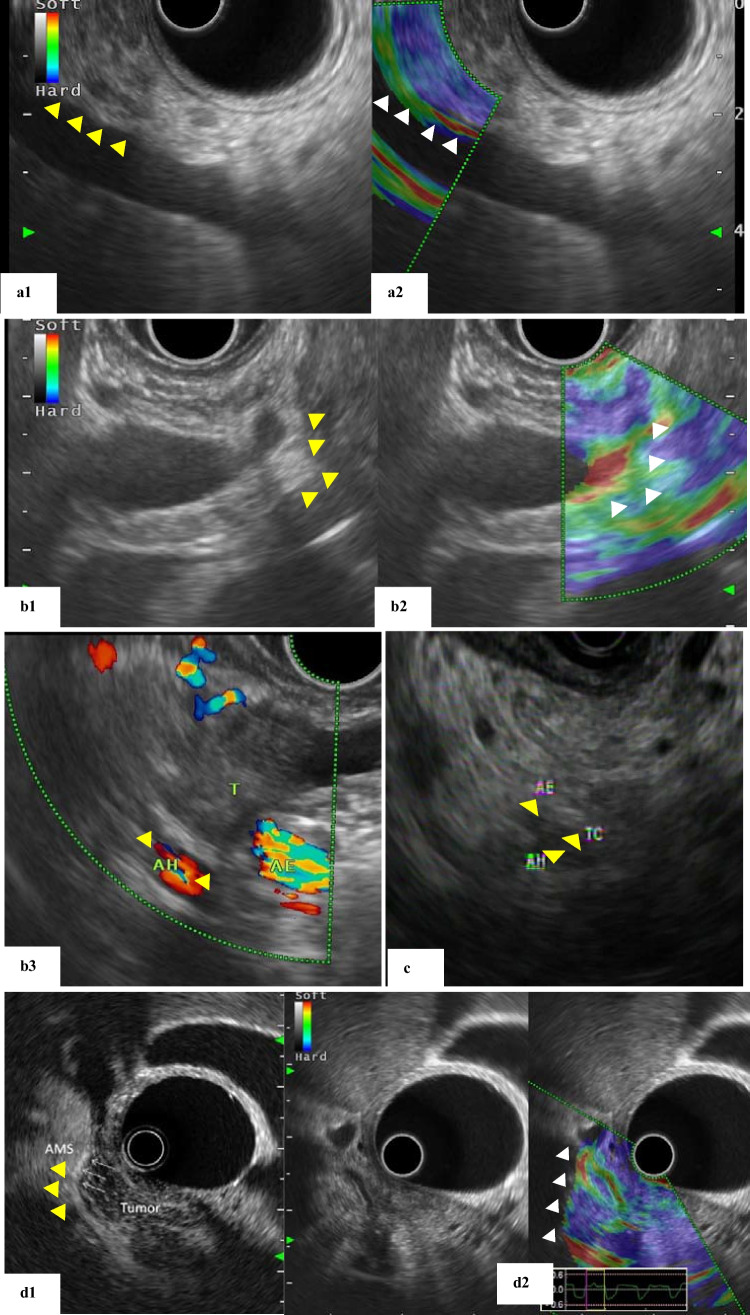
Diagnosis of disruption of the plane III of Inoue by endoscopic ultrasonography and elastography (EUS-EG). **a1**, **b1**, **c**, and **d1** EUS-B mode image with hyperechoic line with no disruption (yellow arrows). **a2**, **b2**, and **d3** EUS-EG image clearly depicts no disrupted plane and green line-low echo area (white arrows). **b3** EUS-B Doppler mode with hyperechoic plane and no VI margin. **d2** EUS-B mode with unclear tumour involvement

The median time of the procedure was 43.5 min (range: 40–60 min). In all cases, the integrity of level III of Inoue was corroborated by EUS-EG, visualising the integrity of the hyperechoic line between the vessel and the tumour and a green line in the elastography views in all the cases, compatible with a low echo area at this level (Fig. 2). The arterial encasement grades were similar to those reported by the CT scan. No occlusion or irregularity was reported. There were no potential side effects after the procedure in all patients. All patients were discharged the same day after the procedure.

### Surgical findings

**Fig. 3:**
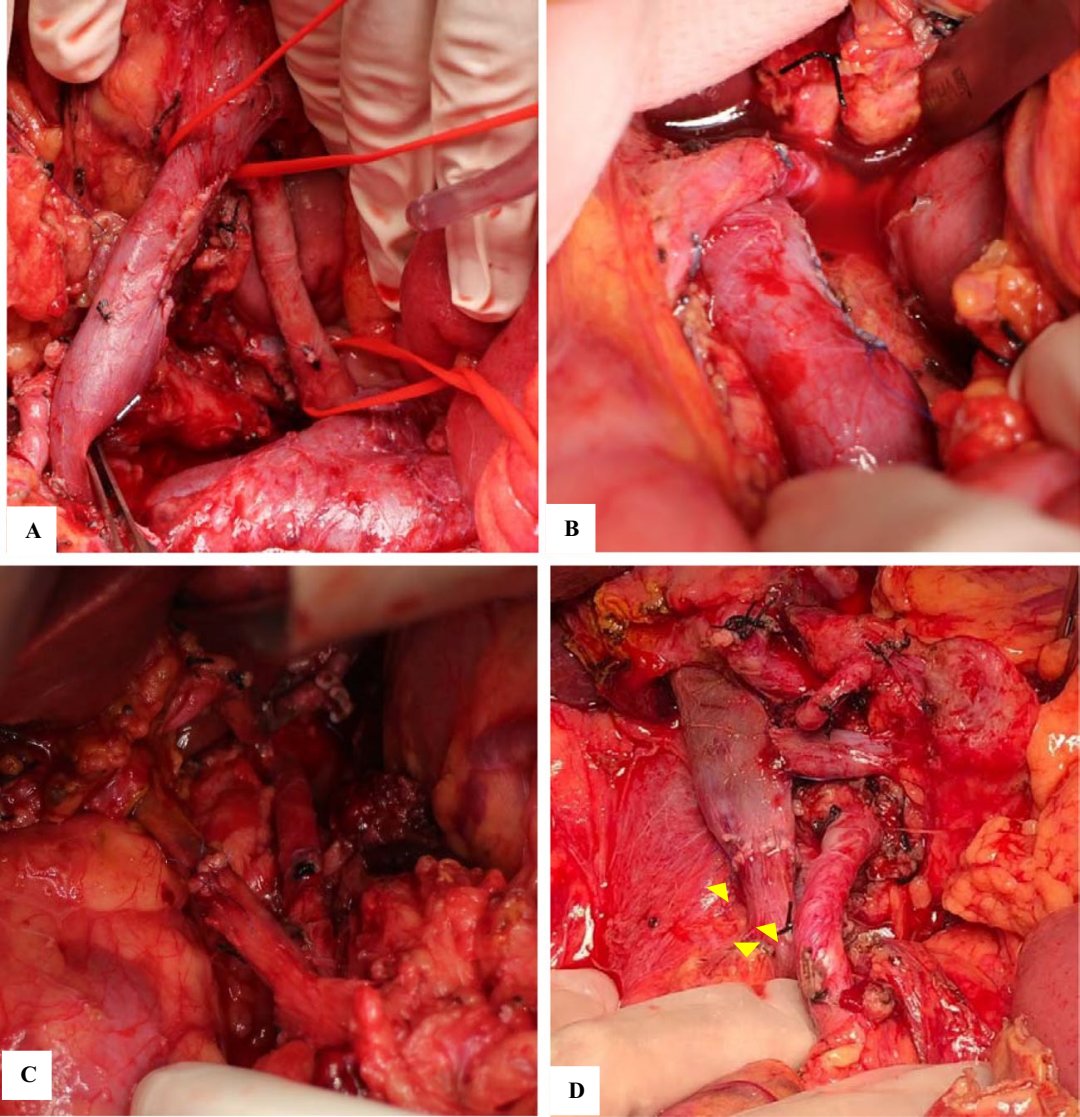
Intraoperative results after resection. Case **A**: tangential superior mesenteric vein resection with peritoneal patch reconstruction. Case **B**: tangential splenic vein—portal confluence resection. Case **C**: triangle operation and porto-mesenteric vein resection with T-T anastomosis. Case **D**: porto-mesenteric vein resection with TT-anastomosis and porto-splenic vein TL anastomosis

All patients were explored by the artery-first approach technique. Of the different artery first approaches described [4, 12], the posterior approach was followed in 3 patients. In every case, Inoue’s level III was reached, and a periarterial divestment was possible to perform in all cases (Fig. 3). In three patients with SMA involvement of fewer than 180°, a right semicircle dissection of the SMA was performed to preserve neurovascular autonomic plexus and avoid uncontrollable postoperative diarrhoea. In one case with SMA > 270°, a complete circumferential resection of the mesopancreas at this level was necessary to complete specimen resection (Table 2).

**Table 2:** Surgical aspects

Case	Tumour location	Surgery	Arterial approach	Inoue plane	Surgical op. time (min)	Blood loss (ml)	Need for transfusion	Arterial resection	Venous resection (type of reconstruction)	Vascular flow (ml/min)		Pancreas consistency
										Artery	Vein	
A	Head-uncinated	PD	Posterior/inferior	III	540	1000	None	No	Tangential SMV (peritoneal patch)	Hepatic (245)	Portal (1.200)	Hard
B	Body	DP	Anterior	III	270	80	None	No	Tangential SV portal confluence (primary raffia)	Hepatic (270)	Portal (1000)	Soft
C	Head-uncinated	TP	Posterior	III	600	2000	Yes (2)	No	Porto-mesenteric resection (T-T anastomosis)	Doppler +	Doppler +	Soft
D	Head-uncinated	PD	Posterior/inferior	III	555	900	None	No	Porto-mesenteric and porto-splenic resection (T-T and LT anastomosis)	R. Hepatic (900)	Portal (900)	Hard

*PD*, pancreaticoduodenectomy; *DP*, distal pancreatectomy; *TP*, total pancreatectomy; *SMV*, superior mesenteric vein; *SV*, splenic vein

Simultaneous complex venous vascular resection was needed in all cases, with a venous patch from the peritoneum in one.

### Histopathological findings

**Fig. 4:**
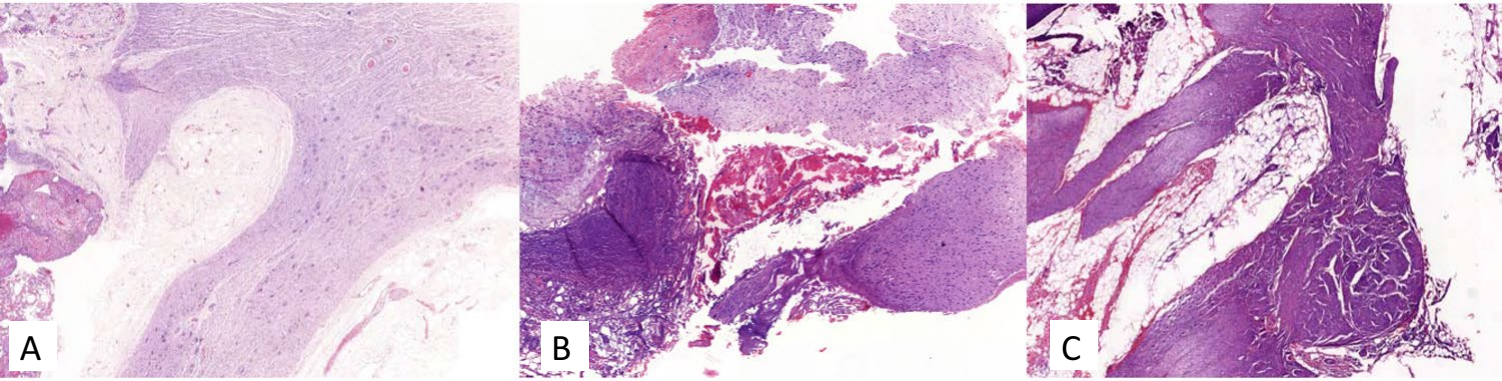
Histopathological samples of periarterial tissue, showing neural hypertrophy and oedema in cases **A**, **B** and **C**

Surgical margins (R0 ≥ 1 mm) were achieved for all patients. The analysis of the anatomopathological specimens focused on the periarterial tissue revealed the presence of neural hypertrophy and small calibre vessels in cases A, B, and D (Fig. 4). Peritumoral fat and small calibre vessel were found in case C. No viable tumoral cells were seen in the arterial margins and periarterial tissue. Those findings are consistent with surgical dissection through the adventitial plane, including the neurovascular plexus around major vessels (AMS, CT, or HA).

## Discussion

This study is the first preliminary report in the literature exploring the EUS-EG as a valuable tool in exploring the integrity of the level III of Inoue, followed by a successful R0 resection in the surgical scenario while adhering to the principles of periarterial divestment, thus avoiding arterial resections.

Recently, neoadjuvant regimes have increased the tumour resection rate in BR and LA pancreatic cancer. FOLFIRINOX is one of the most promising agents, with reported resection rates between 25 and 60% in the best series [3, 13]. The major surgical challenge in BR and LA lies in the arterial encasement by the tumour. Computed tomography predicts resectability with an accuracy of around 95% [14, 15]; however, this falls dramatically after the neoadjuvant treatment, where periarterial stranding makes identifying unresectable disease less accurate with a positive predictive value of only 25% [16]. Previous reports described indirect findings on CT scan suggesting the absence of arterial wall invasion (‘halo sign’) and precluding a periarterial divestment technique [17, 18]. However, this assessment has not been validated in prospective studies. In our cohort, the ‘halo sign’ was not assessed prospectively. Therefore, based on CT scan alone, pre-surgical arterial encasement evaluation and potential resectability, remain problematic, and patients should undergo operative exploration after NT to assess arterial infiltration [10].

On the other hand, the literature did not provide strong evidence favouring a routine arterial resection in pancreatic cancer due to the lack of survival benefit in these patients, the higher mortality rates, and significant postoperative complications [1, 19].

The knowledge of the mesopancreas concept and its levels of dissection has gained interest in the last few years. Inoue described three levels of dissection through the mesopancreas, postulating that level III is the level to perform a free margin and radical surgery in pancreatic adenocarcinoma [4]. Additionally, the concept of periarterial divestment or *periadventitial dissection* technique is only possible when the tumour is divided from the arterial adventitia; this is only possible in cases without infiltration of the tumour through the arterial wall. The periarterial divestment technique has a prominent role in cases of complex LA tumours, avoiding arterial resections and its consequent risk for postoperative complications. In conjunction, the mesopancreas concept and the periarterial divestment technique help surgeons increase the possibility of tumour removal and free margin resections, especially in BR and LA tumours.

Previous reports have explored the role of EUS-EG in predicting resecability [20, 21]. However, none of these reports were approached from the surgeon’s perspective, in which the integrity of level III of Inoue must be found to predict an effective periarterial divestment technique. Furthermore, these reports were conducted in an upfront surgery scenario without considering the effect of neoadjuvant treatment.

The critical aspect that gives EUS-EG an advantage over CT scan in evaluating BR and LA tumours lies in its ability to differentiate ‘soft’ tissues around vessels (identified by a hyperechogenic line or green lines in EG) from solid areas (identified by hypoechoic areas or blue lines in EG). This ability is essential in planning a periarterial divestment technique.

In this regard, the Dutch Pancreatic Cancer Group has conducted a study on intra-operative ultrasound (IOUS) in the context of LA pancreatic cancer following neoadjuvant treatment with FOLFIRINOX, showing an additional value of IOUS in determining vascular involvement and has been used for other tumours to determine resectability during surgical exploration with promising results. In this multicentre study, IOUS was helpful during surgical exploration of LA pancreatic cancer after FOLFIRINOX chemotherapy as it changed the resectability status based on CT scan in approximately one-third of the patients [22]. It is essential to acknowledge that intra-operative ultrasound requires an exploratory laparotomy, which can be considered a drawback.

In our cohort, the histopathological evaluation of the arterial margins identified neurovascular tissue corresponding to the neurovascular plexus in agreement with the achievement of a periarterial divestment technique. Also, neural hypertrophy was found as a frequent pattern (independent of the use of neoadjuvant chemotherapy); this finding has been reported in pancreatic cancer tumours, more frequently at the peripheral, and is probably explained by the promotion of a ‘neural’ tumoral microenvironment by tumoral cells itself, which also contributes to tumour progression [23, 24].

### Limitations of EUS-EG

Based on our experience, one limitation of EUS-EG is previous surgical procedures, such as exploratory laparotomy with previous arterial wall dissection or previous palliative by-pass surgery, due to oedema, fibrosis, and rigidity of the duodenal wall when introducing the endoscope.

It is crucial to address that EUS-EG is an operator-dependent technique, requiring a highly experienced endoscopist to perform a meticulous evaluation of vessels according to our strategy.

Moreover, the distal common hepatic artery and proper hepatic artery are particularly difficult to assess by endoscopic ultrasound, which is one of the main limitations of the technique.

Another limitation is the longer operative time when performing EUS-EG in this scenario and the need for monitored sedation.

Despite the limitations of this preliminary report, our results are encouraging and introduce preoperative EUS-EG as a novel tool in multimodal surgical planning, which is necessary in complex BR and LA pancreatic cancer cases with arterial involvement. The identification of a hyperechogenic line (EUS) or green lines (EUS + EG) around the arterial wall can give surgeons a potential idea of resectability, enabling the application of periarterial divestment techniques, avoiding arterial resections prior to an exploratory laparotomy. These preliminary results should be validated in large prospective trials.
